# Superior Mesenteric Artery Mycotic Pseudoaneurysm Following Infective Endocarditis in a Patient With Rheumatic Heart Disease: A Case Report

**DOI:** 10.7759/cureus.62772

**Published:** 2024-06-20

**Authors:** Nabil Y Al-Madhwahi, Aref A Al-Hashedi, Haitham M Jowah

**Affiliations:** 1 Department of Vascular Surgery, Faculty of Medicine and Health Science, Sana’a University, Sana’a, YEM; 2 Department of Vascular Surgery, Al-Thawra Modern General Hospital, Sana'a, YEM; 3 Department of Surgery, Faculty of Medicine and Health Science, Sana'a University, Sana'a, YEM

**Keywords:** open surgical repair, computed tomography angiography, superior mesenteric artery, mycotic pseudoaneurysm, infective endocarditis

## Abstract

We present a rare case of a 25-year-old woman with rheumatic heart disease who developed a superior mesenteric artery pseudoaneurysm (SMAPA) following infective endocarditis (IE). Initially, she presented with chest pain, dyspnea, and fever, leading to the diagnosis of IE and severe mitral regurgitation. After six weeks of antimicrobial therapy, she developed persistent abdominal pain. Further evaluation revealed a mycotic SMAPA, which was successfully treated with open surgical repair. Postoperatively, her abdominal pain improved significantly, and she was discharged on postoperative day five. The current case underscores the importance of maintaining a high index of suspicion for mycotic pseudoaneurysms in patients with risk factors, even when they present with nonspecific symptoms. The findings also highlight the critical role of computed tomography angiography (CTA) in accurate diagnosis and preoperative planning. The favorable outcome supports current guidelines for managing mycotic SMA pseudoaneurysms in complex scenarios, emphasizing the need for adherence to established protocols and recommendations.

## Introduction

Visceral artery aneurysms (VAAs) and visceral artery pseudoaneurysms (VAPAs) are defined as aneurysms affecting the celiac, superior, or inferior mesenteric arteries and their branches [1]. Aneurysms involve dilation or bulging of the arterial wall, which can lead to life-threatening complications if ruptured [2]. Superior mesenteric artery pseudoaneurysms (SMAPAs) are a particularly rare type, accounting for only 5.5% of all visceral artery aneurysms [3]. Diagnosis of these aneurysms can be challenging due to their often asymptomatic nature, which can result in delayed treatment and an increased risk of severe outcomes, such as intestinal infarction or massive hemorrhage [4,5]. We report a unique case of superior mesenteric artery pseudoaneurysm in a young woman with rheumatic heart disease and a history of infective endocarditis (IE). This case report was previously posted to the Research Square preprint server on April 12, 2024.

## Case presentation

A 25-year-old Yemeni woman with a history of rheumatic heart disease presented to the emergency department with a one-week history of chest pain, dyspnea, and fever. Chest pain was described as sharp and intermittent, predominantly located in the retrosternal area, and was aggravated by deep breathing. The dyspnea was progressive, worsening over the week, and accompanied by a high-grade fever reaching up to 39 °C.

Upon admission, a thorough physical examination was performed. Cardiac auscultation revealed a loud holosystolic murmur at the apex that radiated to the axilla, indicating mitral regurgitation. The initial diagnostic workup included a blood culture, which was positive for Streptococcus viridans, confirming IE. Echocardiography revealed severe eccentric mitral regurgitation (grade IV/IV), thickened and restricted mitral valve leaflets with oscillating masses suggestive of infective vegetation, a dilated left atrium, mild left ventricular dilation, and secondary severe tricuspid regurgitation. A moderate pericardial effusion was also noted.

The patient was treated with intravenous antimicrobial therapy for six weeks, targeting the identified pathogen. After antimicrobial therapy, her chest pain and dyspnea significantly improved. However, one week after treatment, she developed a gradual onset of vague abdominal pain. The pain was initially mild, intermittent, and poorly localized; it was intensified by eating and was alleviated by analgesics. Given the new onset of abdominal pain, a physical examination was repeated, which revealed mild pallor but was otherwise unremarkable. Laboratory studies, including complete blood counts, liver function tests, and assessments of inflammatory markers, were initially unremarkable. Abdominal ultrasonography was performed but was non-diagnostic, so the patient was treated conservatively.

**Figure 1:**
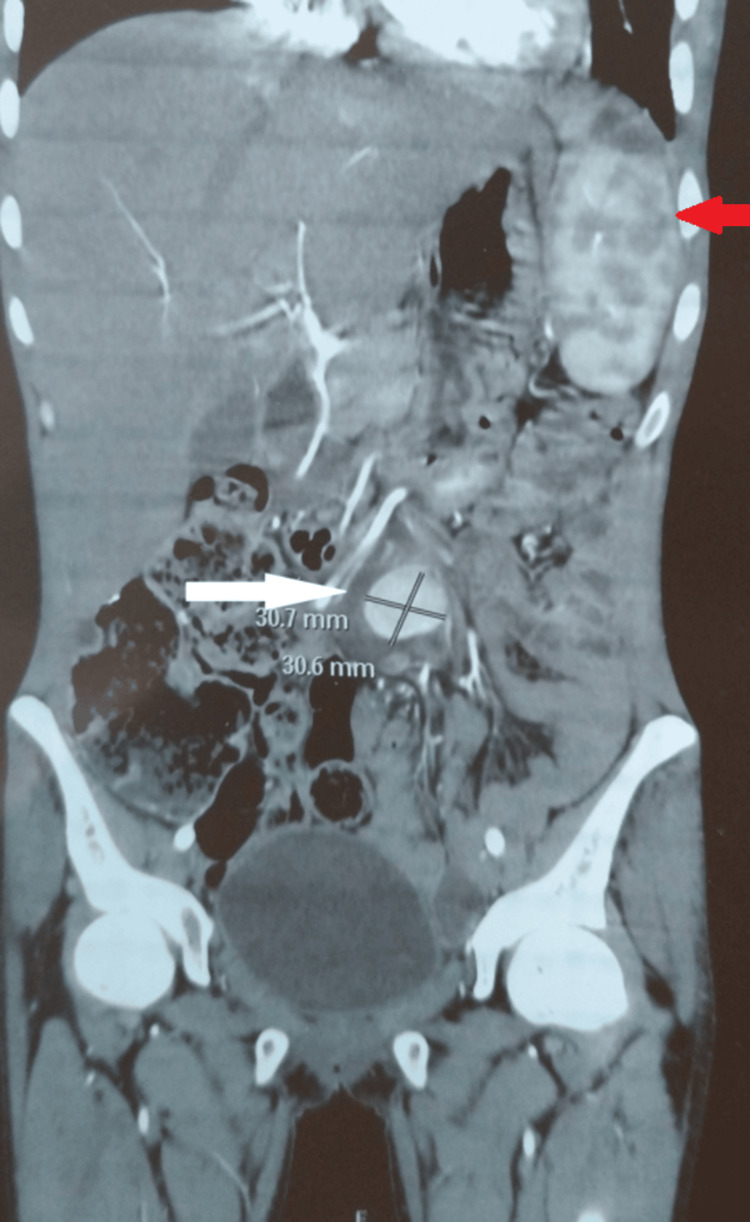
An abdominal CT scan with intravenous contrast (coronal view) shows abnormal aneurysmal dilation of the superior mesenteric artery filled with contrast (white arrow), along with multiple splenic infarctions (red arrow).

**Figure 2:**
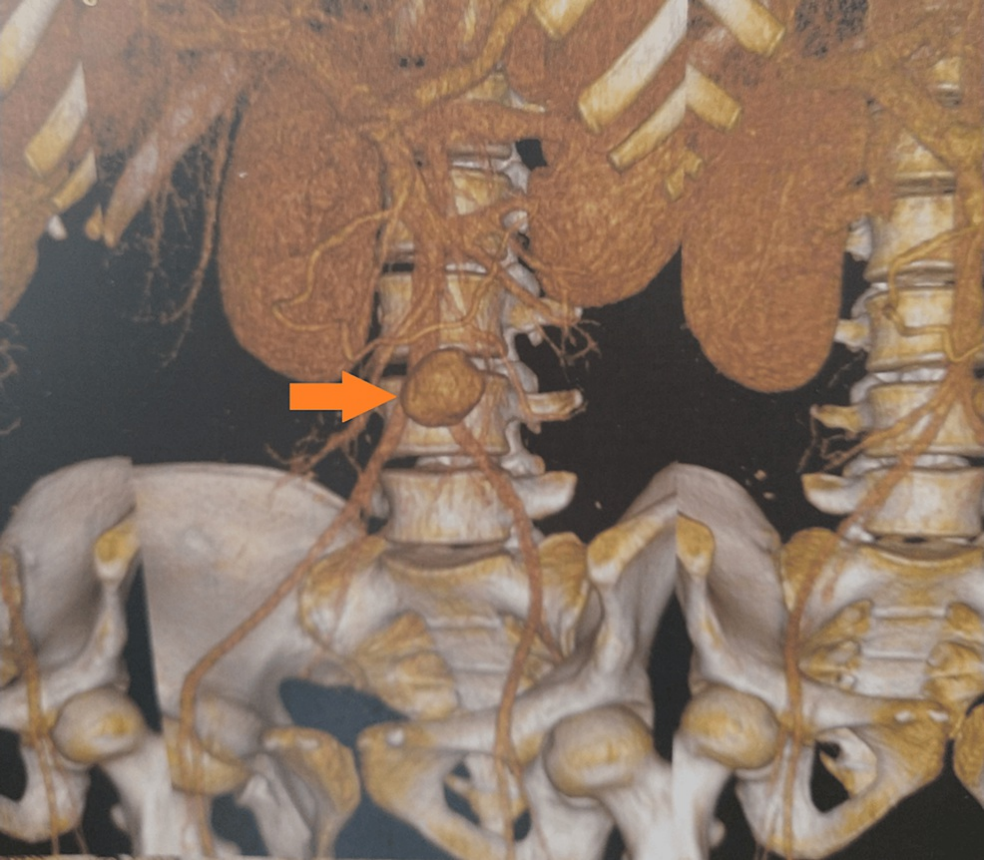
Abdominal CT angiography (3D reconstructive image) shows a pseudoaneurysm in the superior mesenteric artery located 9 cm distal to its origin (orange arrow).

**Figure 3:**
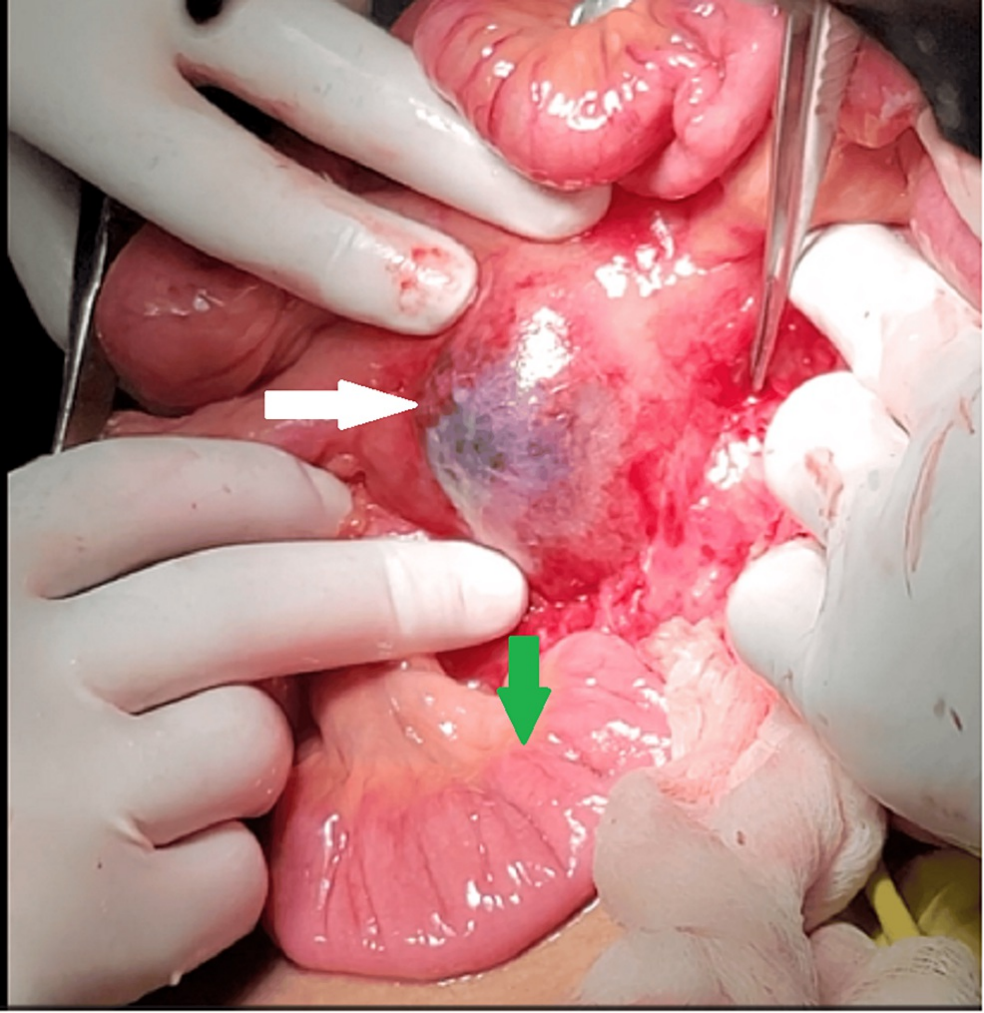
An intraoperative image shows a partially thrombosed pseudoaneurysm of the superior mesenteric artery in the root of the mesentery (white arrow) and healthy small bowel (green arrow).

**Figure 4:**
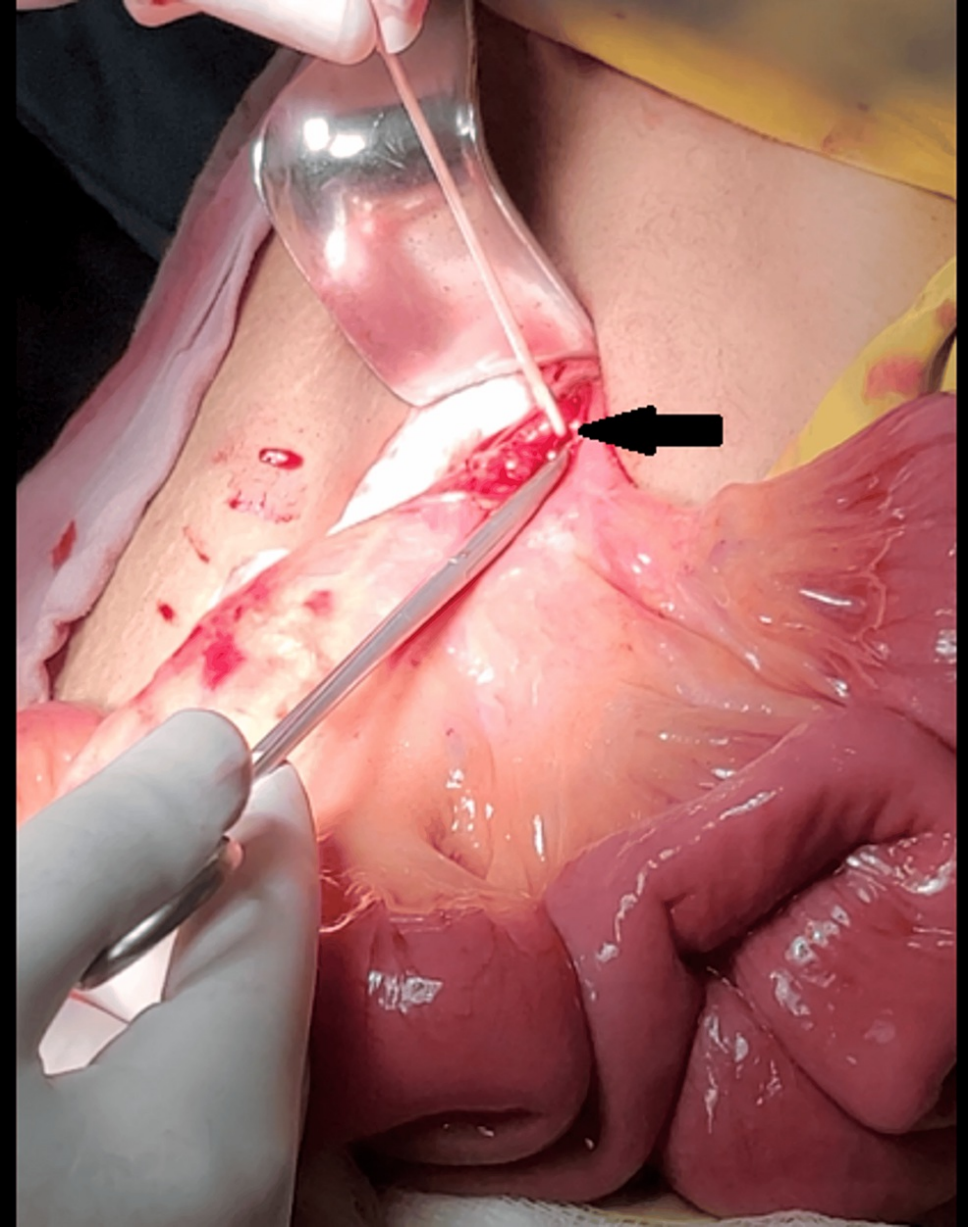
An intraoperative image shows the proximal control of the superior mesenteric artery (black arrow).

**Figure 5:**
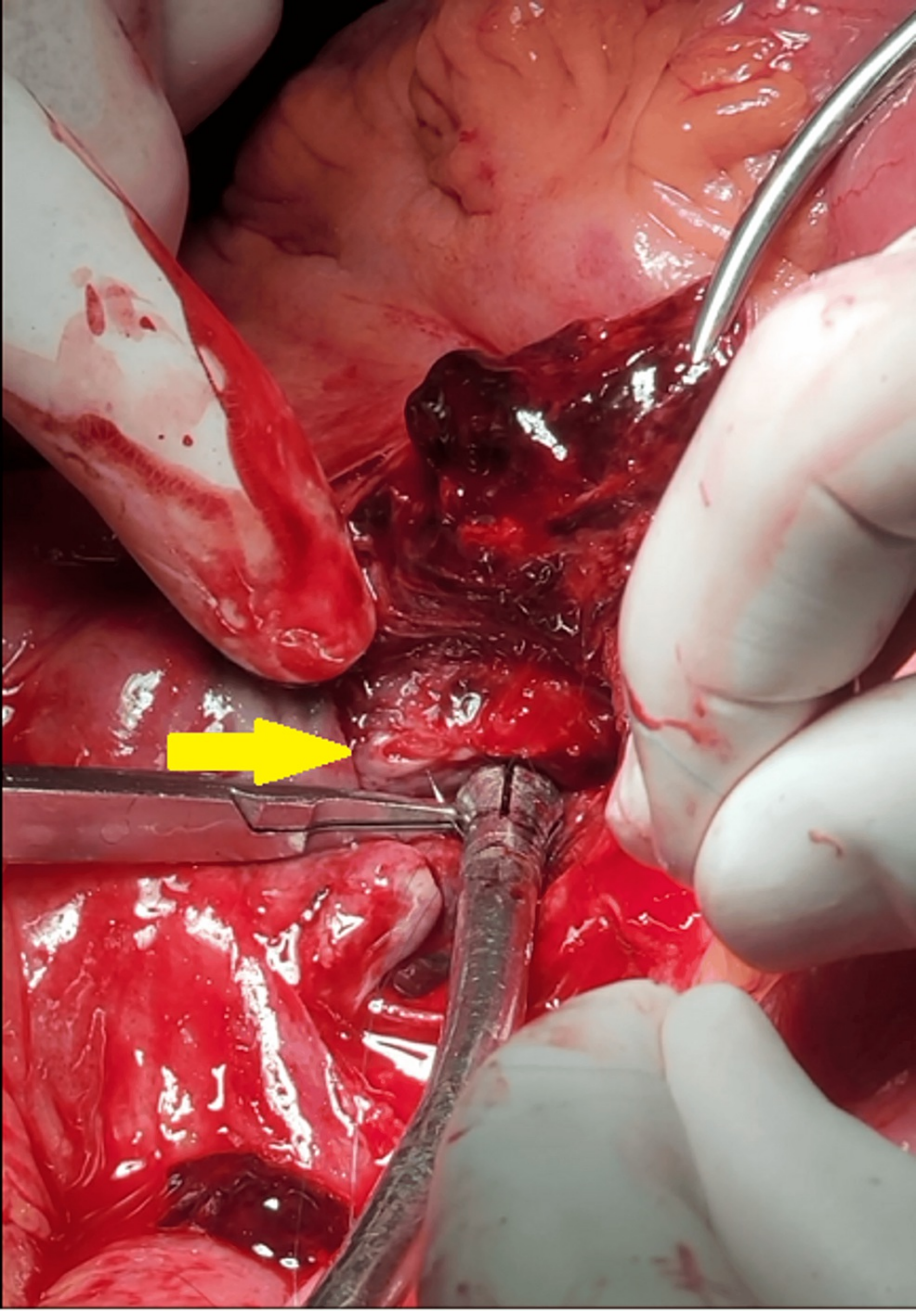
This intraoperative photograph depicts the repair of the defect in the superior mesenteric artery wall using a proline 7-0 suture following the evacuation of pseudoaneurysms (yellow arrow).

During this time, cardiac surgeons decided to perform open mitral valve replacement (MVR) and vegetation resection. The surgery was successful, as indicated by postoperative echocardiography findings, which revealed that the mechanical mitral valve prosthesis was well-seated with good function and that there was improvement in cardiac dimensions and function. The previously severe mitral and tricuspid regurgitation had been resolved. However, one week after cardiac surgery, her abdominal pain intensified, becoming constant and more localized to the periumbilical region, prompting a computed tomography (CT) scan of the abdomen. The CT scan demonstrated a superior mesenteric artery aneurysm (SMAA) 9 cm distal to its origin, measuring approximately 32 mm × 30 mm × 30 mm, with infarctions in the spleen and left kidney due to emboli in the renal and splenic arteries (Figure 1). CT angiography was then performed, which confirmed the presence of a superior mesenteric artery pseudoaneurysm (Figure 2). Subsequently, after a multidisciplinary team discussion, the decision was made to proceed with urgent open surgical repair via laparotomy to avoid possible life-threatening complications, such as mesenteric ischemia, thrombosis, and rupture with massive bleeding. Intraoperative findings revealed a partially thrombosed pseudoaneurysm at the root of the mesentery with a healthy small bowel (Figure 3). The pseudoaneurysm was successfully resected after proximal and distal control of the SMA (Figure 4), and the arterial wall was repaired using a proline 7-0 suture (Figure 5).

The postoperative course was uncomplicated, with significant improvement in abdominal pain. She was discharged on postoperative day 5 in stable condition.

## Discussion

SMAAs and SMAPAs are rare but critical vascular conditions that are often associated with significant morbidity and mortality because of their potential to rupture. In this case, the pseudoaneurysm was mycotic, a complication of IE. This report underscores several crucial aspects of the diagnosis and management of complex vascular lesions.

SMAAs and SMAPAs are visceral arterial aneurysms that can be mycotic, are often associated with infective endocarditis, and present with nonspecific symptoms [6]. These conditions include abdominal pain, which can be intermittent or severe; a throbbing mass in the abdomen; shock; and, in rare cases, obstructive jaundice. However, they can also be initially asymptomatic, leading to a delayed diagnosis [7,8]. Early diagnosis and treatment are crucial for reducing the risk of intestinal infarction and mortality and preventing life-threatening complications such as mesenteric ischemia, thrombosis, and rupture with massive bleeding [9,10]. The rupture of pseudoaneurysms can have severe consequences, with mortality rates ranging from 25% to 70% [11].

The diagnosis of mycotic aneurysms and pseudoaneurysms is based on a combination of clinical symptoms, imaging findings, and microbiological culture results, if available. Given the lack of specific symptoms, a high index of suspicion is needed in patients with risk factors such as intravenous drug use, prosthetic valves, and other conditions that predispose them to infection [12]. Computed tomography angiography (CTA) is the recommended diagnostic tool of choice for mycotic pseudoaneurysms because it allows enhanced visualization of the lesion [13,14]. Contrast-enhanced CT revealed the pseudoaneurysm as an enhanced lesion. Mesenteric angiography, although less commonly used than computed tomography, can provide a detailed visualization of the vasculature, help confirm the diagnosis, and aid in preoperative planning for SMA aneurysm repair [14]. These imaging techniques play a vital role in the timely and accurate diagnosis and preoperative planning of mycotic SMA pseudoaneurysm repair. The diagnosis of mycotic SMAPA in this patient highlights the importance of maintaining a high index of suspicion in patients presenting with nonspecific symptoms, particularly those with underlying conditions like rheumatic heart disease and recent IE. The initial presentation of vague abdominal pain exacerbated by eating posed a diagnostic challenge because initial laboratory tests and ultrasonography were non-diagnostic.

Treatment indications are generally recommended for all identified SMAA/SMAPA, regardless of lesion size or presence of symptoms, according to recently published clinical practice guidelines on the management of VAA/VAPA [14]. The choice of treatment depends on various factors, such as the specific anatomy of the aneurysm, the expertise of the medical team, the overall condition of the patient, and the presence of infection as the cause of the aneurysm [14]. Surgery remains the mainstay of treatment for mycotic pseudoaneurysms. Resection of the infected tissue and subsequent vascular reconstruction using an autogenous conduit are preferred [5]. However, vascular reconstruction via aneurysmorrhaphy or ligation has been reported [15,16]. On the other hand, endovascular techniques, including coiling, glue embolization, and the placement of covered stents, may also be used in some cases, with the recommendation of preoperative broad-spectrum antibiotics [17].

In our particular case, a 25-year-old woman with rheumatic heart disease was diagnosed with infective endocarditis and severe mitral regurgitation. After six weeks of antimicrobial therapy, her symptoms improved, but she developed abdominal pain. Despite the initial nondiagnostic workup, the patient underwent mitral valve replacement and vegetation resection. Postoperatively, her pain worsened, and imaging revealed SMAPA and infarctions in the spleen and kidney. Due to the pseudoaneurysm's location and infectious etiology, open surgical repair was performed, resulting in the successful resection and repair of the SMAPA. The postoperative course was uncomplicated, with significant improvement in pain. The current case underscores the importance of maintaining a high index of suspicion for mycotic pseudoaneurysms in patients with risk factors, even when they present with non-specific symptoms. The findings also highlight the critical role of gold-standard imaging in accurate diagnosis and preoperative planning. The favorable outcome supports current guidelines for managing mycotic SMA pseudoaneurysms in complex scenarios, emphasizing the need for adherence to established protocols and recommendations [5,14].

## Conclusions

Mycotic SMA pseudoaneurysms are rare but life-threatening complications of infective endocarditis, particularly in patients with underlying heart disease. Early diagnosis and treatment are crucial for preventing intestinal infarction and mortality. CTA plays a vital role in the timely and accurate diagnosis of mycotic SMA pseudoaneurysms. Surgical resection of the infected tissue and subsequent vascular reconstruction are the mainstays of treatment for mycotic pseudoaneurysms. This case underscores the importance of maintaining a high clinical suspicion of mycotic pseudoaneurysms in patients with risk factors and the use of CTA as the gold standard imaging modality for accurate patient evaluation.
